# Predictors of Condom Use Behaviors Based on the Health Belief Model (HBM) among Female Sex Workers: A Cross-Sectional Study in Hubei Province, China

**DOI:** 10.1371/journal.pone.0049542

**Published:** 2012-11-20

**Authors:** Jinzhu Zhao, Fujian Song, Shuhua Ren, Yan Wang, Liang Wang, Wei Liu, Ying Wan, Hong Xu, Tao Zhou, Tian Hu, Lydia Bazzano, Yi Sun

**Affiliations:** 1 Department of Social Medicine and Health Management, School of Public Health, Tongji Medical College, Huazhong University of Science and Technology, Wuhan, Hubei Province, China; 2 Norwich Medical School, Faculty of Medicine and Health Science, University of East Anglia, Norwich, United Kingdom; 3 Department of Epidemiology, School of Public Health and Tropical Medicine, Tulane University, New Orleans, Louisiana, United States of America; Tulane University, United States of America

## Abstract

**Background:**

HIV infection related to commercial sexual contact is a serious public health issue in China. The objectives of the present study are to explore the predictors of condom use among female sex workers (FSWs) in China and examine the relationship between Health Belief Model (HBM) constructs.

**Methodology/Principal Findings:**

A cross-sectional study was conducted in two cities (Wuhan and Suizhou) in Hubei Province, China, between July 2009 and June 2010. A total of 427 FSWs were recruited through mediators from the ‘low-tier’ entertainment establishments. Data were obtained by self-administered questionnaires. Structural equation models were constructed to examine the association. We collected 363 valid questionnaires. Within the context of HBM, perceived severity of HIV mediated through perceived benefits of condom use had a weak effect on condom use (*r* = 0.07). Perceived benefits and perceived barriers were proximate determinants of condom use (*r* = 0.23 and *r* = −0.62, respectively). Self-efficacy had a direct effect on perceived severity, perceived benefits, and perceived barriers, which was indirectly associated with condom use behaviors (*r* = 0.36).

**Conclusions/Significance:**

The HBM provides a useful framework for investigating predictors of condom use behaviors among FSWs. Future HIV prevention interventions should focus on increasing perceived benefits of condom use, reducing barriers to condoms use, and improving self-efficacy among FSWs.

## Introduction

By the end of 2009, there had been 560,000–920,000 persons are infected with HIV in China, and the cumulative number of deaths due to HIV had reached 200,000–240,000 [1]. Although intravenous drug use and commercial blood/plasma collection used to be the main causes of the HIV infection in China, heterosexual transmission of HIV become increasingly important in recent years. The heterosexual transmission accounted for 50% of HIV infection in 2005, 57% in 2007, and 75% in 2009 [2], [3].

### Female sex workers (FSWs) and HIV in China

One of the factors that drive the rapid spread of HIV in Asia is the infection transmitted between sex workers and their clients, which is then spread to the general population [4]. It is difficult to have a precise estimate of the number of female sex workers (FSWs) in China [5]. One widely cited estimate of the number of FSWs in China lies between 4 and 10 million [6], although the actual number may be higher. The prevalence of HIV among FSWs has increased dramatically from 0.03% in 1995 to 0.57% in 1998 and to 1.37% in 2005 [7]. According to a report in 2007, the prevalence of HIV among FSWs was on average about 1% [8]. Data from the national sentinel surveillance revealed that the rate of HIV infection was as high as 10.3% in Kaiyuan city of Yunnan province, and 16% in some areas of Guangxi province, mainly due to the high prevalence of both drug abuse and unsafe sexual contacts [9], [10], [11], [12]. Compared with the general population, the HIV prevalence is up to 20 times higher among FSWs [13]. There were approximately 127,000 FSWs and their clients living with HIV/AIDS in 2005 in China, accounting for 20% of the total diagnosed HIV cases [3].

China began economic reform since 1980s, and the re-emergence of illegal sex industry in China is one of the unwanted by-products of a market economy. In addition, the resurgence of economy has also led to changes of attitudes and sexual behaviors in the general population in China [6]. Consequently, the incidence of sexually transmitted diseases (STDs) increased on average by 39.5% per year between 1985 and 2000, and the annual cases increased by 147 fold during the same time period [6], [14].

In most urban and suburb area of China, FSWs exist in a more or less open way. Commercial sex takes place in many settings including luxury hotels, small guesthouses, roadside stops of trucks and various entertainment establishments (e.g., dance halls, karaoke bars, massage parlors, bathhouses, hair-washing rooms). Because commercial sex is illegal in China, spot checks are often carried out by enforcement officers of government. If FSWs are arrested, they are subject to fines and incarceration according to the law [6].

There are considerable differences among FSWs in China, in terms of workplaces and sexual health risks [15], [16], [17]. FSWs at the “high-tier” entertainment workplace (e.g. luxury hotels or night clubs) charge much and usually work in private settings while those at the “low-tier” workplace (e.g. hair solon or massage rooms) charge little and can be accessed by a wider client base [18]. Evidence suggests that FSWs at “low-tier” workplace are more vulnerable to the infection of AIDS, syphilis or other STDs [19], [20].

### Condom use of FSWs in China

It is reported that consistent and correct condom use can reduce the risk of HIV infection in FSWs by approximately 69% [21]. The proportion of condom use in FSWs has been increasing in China [9], [22], [23], [24], [25], however, data from a national surveillance in 2007 indicated that 60% of Chinese FSWs still do not use condoms regularly [2].

Condom use by FSWs may be affected by individual characteristics (e.g., knowledge, beliefs, skills, self-efficacy, drug abuse, alcohol), working conditions (e.g., sexual partners, gatekeepers, workplace, community and culture), and socioeconomic factors (e.g., policy, health care systems, law enforcement and legislature). It is important to understand factors that may influence the condom use behaviors in FSWs.

### Health belief model

The HBM was developed to explain the lack of public participation in health screening and prevention programs in the 1950s [26]. It has been widely used to explore a variety of health behaviors including sexual risk behaviors and the transmission of HIV/AIDS [27]. The key variables of the HBM include the severity of a potential illness, the person's susceptibility to the illness, the benefits of taking a preventive action, and the barriers to taking that action, cues to action, and self-efficacy. Benefits and barriers are usually strong predictors of the relevant behavior, whereas susceptibility and severity may not [28], [29].

Findings from previous research on predictors of consistent condom use in FSWs were contradictory. Some research showed that perceived susceptibility to STDs/HIV, the perceived benefit and self-efficacy were important determinants of consistent condom use [22], [30], [31], [32]. However, studies conducted in Indonesia among FSWs found that perceived susceptibility and severity to STDs/HIV, and self-efficacy were unrelated to the behavior of consistent condom use [33], [34]. A few behavioral studies have been conducted among FSWs in China. A survey of 454 establishment-based FSWs in a rural county of Guangxi Province reported that the use of condom in FSWs was not associated with perceived susceptibility and severity, and negatively associated with perceived benefits of condom use [31]. A cross- sectional study conducted in Jinan City of Shandong Province reported that the use of condom in FSWs was directly associated with self-efficacy, which was consistent with the study in Guangxi Province [22]. Controversial results from previous studies make it necessary to conduct further research on the predictors of condom use behavior. The objective of this study were to investigate predictors of condom use behaviors in FSWs and to examine association between different variables.

## Methods

### Study site

This study was conducted in Wuhan and Suizhou of Hubei Province between July 2009 and June 2010. Wuhan is the capital city of Hubei Province with 9.79 million residents [35]. By the end of 2009, there had been 1,600 patients with HIV/AIDS in Wuhan [36], accounting for 29% of the reported cases in Hubei Province [37]. Sexual contact is the major transmission route in Wuhan, and 60% of people living with HIV/AIDS (PLHIV) contracted HIV through sexual contact [38]. In contrast, Suizhou is located in the north of Hubei Province with 2.16 million residents [35]. By the end of 2009, the total number of cases attributed to HIV/AIDS in Suizhou was 643, accounting for 12% of the reported cases in Hubei Province [39], [40]. Most HIV infections in Suizhou were caused by commercial blood/plasma collection before 2006, but sexual contact has become the dominant transmission since 2007. In 2007–2009, it was reported that 61% of people living with HIV/AIDS were infected by sexual contact in Suizhou [39].

### Recruitment of seeds and study participants

The study included women who were working at ‘low-tier’ entertainment establishments (e.g. street, rental houses, hair salons, sauna rooms, karaoke bars, massage parlors, and small hotels). Those who self-reported charging sexual activity during the last six months were included in this study. Written informed consent forms were obtained from all participants involved in this study. The study protocol has been approved by the Institutional Review Board at Tongji Medical College, Huazhong University of Science and Technology.

FSWs are usually difficult to reach because commercial sex is clandestine [41]. Thus, we recruited coordinators from a Non-Government Organization that provides support to the research on FSWs in Wuhan and a volunteer who is familiar with the commercial sex industry in Suizhou. The study recruited a total of 427 FSWs, 218 in Wuhan and 209 in Suizhou.

With the permission, one-to-one interviews were carried out in private rooms or spaces. Four coordinators were trained in the School of Public Health of Huazhong University of Science and Technology. A coordinator provided the consent form to each person in the source population and informed that the participation was completely voluntary, anonymous and confidential. If the person would like to participate in the study, one was asked to fill in the questionnaire. In most cases, the questionnaire was self-administered by participants. For those who had inadequate literacy, the coordinator read through the questionnaire and completed it according to participants' responses. The coordinator checked and corrected the questionnaire for any omission or unidentified responses. Each participant took 20–30 minutes for interview and received an incentive package which was worth about 50 Yuan (U.S. $ 7.85), including four boxes of condoms (40 condoms), AIDS prevention educational manuals, and some household items such as towels, wet wipes, and soap. Participants did not complete the questionnaire because of various reasons such as the interruption by their clients or FSWs becoming impatient, and those who did not complete the questionnaire were excluded. We finally collected 363 completed questionnaires.

### Measures

We developed a structured questionnaire and tested in a pilot study among 20 FSWs before the present study. The questionnaire included information on demographics (such as age, education levels, and marital status), job information, drug abuse, abortion history, condom use behaviors, and the constructs of the HBM model (perceived susceptibility, perceived severity, perceived benefits, perceived barriers, and self-efficacy).

Among the constructs of the HBM model, perceived susceptibility and severity, or health threat, was defined as the perceived risk and unwanted consequences of HIV infection. Perceived susceptibility was measured with 2 items about individual's subjective perception of the risk of the threat (e.g., Do you think you are at the risk of HIV infections?). The internal consistency estimate (Cronbach's α) for this 2-item scale was 0.45. Perceived severity was measured with 3 items about perceived seriousness of contracting an illness or of leaving one untreated (e.g., If I had AIDS, my life will end soon.). This 3-item scale had a Cronbach's alpha coefficient of 0.58. Answer options were 1 (disagree), 2 (neutral attitude), and 3 (agree). A higher score indicated a higher level of perceived susceptibility/severity.

Four items were used to assess the believed effectiveness of condom use at reducing the threat of STDs/AIDS or pregnancy. Cronbach's alpha coefficient for this 4-items scale was 0.75. Answer options were 1 (disagree), 2 (neutral attitude), and 3 (agree).

Perceived barriers to condom use during commercial sex were assessed by four items. Two items were used to measure the availability of condoms. Typical items included “inconsistent condom use can save money.”, and “when you want to use condoms, they are just not around”. Two items were used to measure perceived barriers due to clients' demand (e.g., if a client paid more for having sex without using condom, would you agree to do so?). Cronbach's alpha coefficient for this 4-items was 0.63. Answer options were 1 (disagree), 2 (neutral attitude), and 3 (agree).

Self-efficacy of condom use was measured using a three-item scale assessing the confidence of using condoms with clients (e.g., if a client was unwilling to use condoms, could you convince him to use condoms?”). Response options were 1 (No), 2 (neutral attitude), and 3 (Yes). The internal consistency estimate (Cronbach's α) for this 3-item scale was 0.46.

In this study, condom use behavior was defined as the use of a condom with a client during vaginal intercourse. It was assessed for the last sex episode and the past month prior to baseline assessment. Two items were measured with different response categories (“no”/“yes” and “never”/“sometimes”/“always”,respectively) and the Cronbach's alpha coefficient for the 2-items scale was 0.67.

## Data analysis

**Figure 1:**
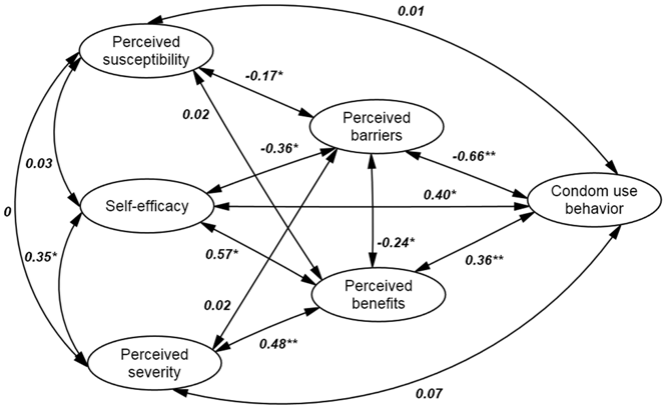
Structural equation model depicting regression paths in the HBM model (N = 363). The ovals represent latent variables, and double-headed arrows represent correlations. Path coefficients were shown, and 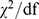 = 273.6/120 = 2.28, CFI = 0.91, GFI = 0.93, RMSEA = 0.05. **P<0.001, *P<0.05.

**Figure 2:**
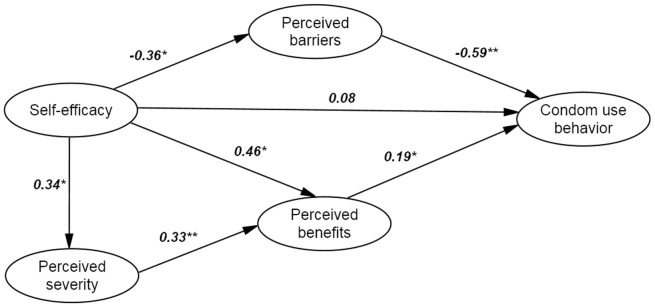
The initial hypothesized model. Path coefficients were shown above. 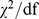 = 193.9/97 = 2.0, CFI = 0.93, GFI = 0.94, RMSEA = 0.05. **P<0.001, *P<0.05.

**Figure 3:**
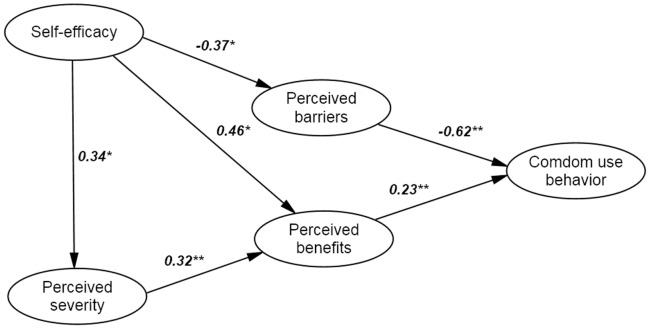
The final model. Path coefficients were shown above. 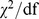 = 193.9/97 = 2.0, CFI = 0.93, GFI = 0.94, RMSEA = 0.05. **P<0.001, *P<0.05.

Descriptive statistics, such as Frequencies, percentages, means and standard deviations of baseline variables were calculated to describe the characteristics of the sample. Structural equation model (SEM) was used to analyze the proposed model structure of HBM through the factor analysis and path analysis. The SEM process includes the following steps. First, confirmatory factor analysis (CFA) was performed to examine whether there is empirical support for the proposed theoretical factor structure, and the relationships among latent variables (e.g., perceived severity) and manifest (observed) variables. Standardized regression weights were generated (see Figure 1) in these models and Cronbach's alpha was calculated for the internal consistency among variables from each latent construct. Based on the confirmed factor structure, a path model was then built to identify significant predictors associated with condom use and potential relationships among all latent variables (see Figure 2). Finally, a parsimonious model was generated by using a model-fitting in which non-significant paths were dropped gradually (see Figure 3) [42].

The maximum likelihood method of parameter estimation was used in the SEM analysis, and goodness-of-fit tests were conducted by using absolute and comparative fit indices, including 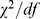, the root mean square errors of approximation (RMSEA), goodness of fit index (GFI), and comparative fit index (CFI). The model fit was assessed according to the following criteria: 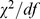
<3, RMSEA<0.08, GFI>0.90, and CFI>0.90 [43], [44].

The statistical analyses were performed using SPSS 15.0 (SPSS Inc., Chicago, IL), and Amos 7 (free student version).

## Results

### Characteristics of Study Participants

Table 1 describes participants' demographic characteristics. The mean age of the women was 26.3 years (SD = 6.9). Among the participants, 79.6% received middle school education or less, 60.5% were unmarried, 69.3% had worked as FSWs for 2 years or less, and 66.0% had non-commercial stable sexual partner(s). Most of the participants (74.9%) reported using condoms consistently with clients during vaginal intercourse in the last month and 91.2% in the last episode. Only a small proportion of the participants (18.7%) reported using condoms every time with non-commercial stable sexual partner(s). In addition, 39.9% procured an abortion, 5.5% admitted drug abuse, and 68.0% bought condoms themselves.

**Table 1:** Socio-demographic characteristics of female sex workers (N = 363).

Characteristic	N	%
Age (N = 360)		
≤20 years	82	22.8
20∼30 years	191	53.1
>30 years	87	24.2
Marriage status (N = 362)		
Unmarried without cohabitant	67	18.5
Unmarried with cohabitant	152	42.0
Married and live with husband	87	24.0
Married but not live with husband	52	14.4
Divorced or widowed	4	1.1
Education (N = 363)		
Elementary school	81	22.3
Junior high school	208	57.3
Senior high school and above	74	20.4
Time engaged in job (N = 361)		
≤1 year	183	50.7
1∼2 years	67	18.6
>2 years	111	30.7

### Confirmatory factor analysis

The standardized regression weights, means and standard deviation are shown in Table 2. All standardized regression weights were statistically significant (*P*<0.05), except for those with regard to perceived susceptibility to HIV. Thus, perceived susceptibility against other latent variables was excluded in the subsequent analyses.

**Table 2:** Summary statistics and standardized regression weights in confirmatory factor analyses.

Item		Standardized regression weights							
		BCU	PSu	PSe	PBe	PBa	SE	M	SD
1	Sexual behavior in last month	0.73						2.7	0.4
2	Last sexual behavior	0.75						1.9	0.3
3	Chance of getting HIV (1)		1.41					1.9	0.7
4	Chance of getting HIV (2)		0.21					1.5	0.6
5	Expensive to treat HIV			0.39				2.5	0.7
6	Fear about discrimination			0.65				2.5	0.7
7	Fear about death			0.63				2.2	0.8
8	Avoid getting HIV (1)				0.57			2.6	0.7
9	Avoid getting HIV (2)				0.78			2.5	0.7
10	Avoid getting STDs				0.83			2.6	0.7
11	Contraception				0.46			2.6	0.6
12	Money spending					0.37		1.5	0.8
13	Condom accessibility					0.48		1.6	0.7
14	Request of the guests					0.73		1.3	0.5
15	Extra income					0.69		1.5	0.7
16	Ability of persuade customers						0.14	2.4	0.7
17	Ability of carrying condom						0.80	2.8	0.5
18	Ability of using condom						0.72	2.7	0.6

Note: M = mean, SD = standard deviation, BCU = Behavior of Common Use, PSu = Perceived Susceptibility, PSe = Perceived Severity, PBe = Perceived Benefits, PBa = Perceived Barriers, SE = Self-efficacy;

Except for items in PSu, all standardized regression weights were significant (*P*<0.05),

Figure 1 presents the the goodness-of-fit statistics and it suggested that the linear structural equation model is appropriate for our data (see Figure 1). Figure 1 also shows the paths of the full model and the pair-wise correlation coefficient between variables in the HBM. Condom use was significantly associated with perceived benefits, perceived barriers, and self-efficacy.

### Path models

The initial hypothesized model is shown in Figure 2. There were one exogenous variable (i.e., self-efficacy) and 4 endogenous variables (i.e., perceived severity, perceived benefits, perceived barriers, and condom use). It was assumed that the endogenous variables were influenced by other observed variables, whereas the exogenous was not. The fit indices for the initial model seemed acceptable. To generate a parsimonious model, non-significant paths were eliminated. The final HBM model is depicted in Figure 3, which has the similar fit indices with the previous model and the construct is more compact than that in the initially.

Figure 3 represents the association between potential latent variables. Both higher level of perceived benefits and lower level of perceived barriers were associated with more condom use; but perceived barriers on condom use has a greater r than perceived benefits (0.62 vs. 0.23). Higher level of self-efficacy was significantly related to greater perceived severity, greater perceived benefits, and lower perceived barriers and all these associations were significant. Table 3 represents direct effects, indirect effects, and total effects of variables on condom use. Self-efficacy had an indirect effect on condom use and the correlation coefficient of self-efficacy on condom use was 0.36. Perceived severity was indirectly associated with condom use through perceived benefits with a correlation coefficient of 0.07. The final model accounted for 50% of the variance in condom use.

**Table 3:** Direct and indirect effects on condom use.

	Direct Effects	Indirect Effects	Total Effects
Perceived severity	0.00	0.07	0.07
Perceived benefits	0.23	0.00	0.23
Perceived barriers	−0.62	0.00	−0.62
Self-efficacy	0.00	0.36	0.36

## Discussion

This study demonstrated the utilization of the HBM model for investigation predictors of condom use during commercial sex among Chinese FSWs. The results of this study showed that FSWs who possessed higher levels of perceived severity, perceived benefits, self-efficacy, and lower levels of perceived barriers toward condom use were more likely to use condoms.

Perceived severity of HIV had a positive effect on condom use, but the effect was indirect and weak (coefficient of indirect effects = 0.07). This finding is consistent with several previous studies in which perceived severity was the weak predictor of behaviors [28], [29], although other research showed that perceived susceptibility and severity had a negative indirect effect on condom use in FSWs [31]. One possible explanation of the weak effect and indirect is the association of perceived severity and susceptibility with condom use was mediated by other variables [28], [45], [46], and data in our study supported this hypothesis, indicating that perceived benefits might be the intermediated variable. Greater perceived severity of HIV/STDs results in greater perceived benefits of condom use. This finding is important because it suggests that the perceived severity of HIV/AIDS is necessary but may not be sufficient to improve the behavior of condom use.

Our data represented low level of perceived susceptibility of HIV (mean score of the item<2) and a high level of perceived severity (mean score of the item>2) among FSWs. People may not be motivated to change behaviors when they fail to recognize the severity and susceptibility to the great extent [28]. Therefore, future HIV prevention interventions should continue to devote effort to promoting perceived susceptibility and severity among FSWs. In addition, perceived severity of extremely severe illness might not vary greatly compared to other variables and low variation may also result in low estimates of effect size [47]. However, this possibility is not confirmed by our study.

We found that perceived benefits and barriers had a direct effects on condom use, which is consistent with findings from previous studies [28], [31], [48]. We defined barriers to condom use as condom availability and client demand in our study. We did not consider drug abuse, threats of violence from clients and alcohol consumption as in other studies [11], [49], [50], because we found that clients' requests and extra payment for not using condoms were major barriers. In addition, our results suggest that perceived barriers on condom use was greater than the perceived benefits. Thus, reducing barriers to condom use may be more effective than increasing the awareness of benefits to improve the condom use behaviors. Furthermore, it was shown that 68% of FSWs purchased condoms and these FSWs may not buy those of good quality. Thus, future intervention studies should not only try to reduce barriers to condom use, but also to focused on the improvement in accessing condoms of good quality among Chinese FSWs.

Some previous studies reported that self-efficacy, as one of the most important factors in condom use, had a positive effect on condom use frequency, consistency and intention to use a condom [51], [52], [53], [54], although a few studies did not find any effect of self-efficacy [55], [56], [57]. Self-efficacy of condom use is defined as the belief that one is both capable of and likely to use condoms in sexual activities [56]. One may know how to use a condom, but may not feel confident in their ability to do so [58]. Albert Bandura who proposed self-efficacy theory suggested that, perceived self-efficacy contributes to cognitive development and functioning, and exerts its influence through cognitive, motivational, affective, and selection processes [59]. Songpol Kulviwat suggested that self-efficacy was an important antecedent of the perceived usefulness and ease of use of a new technology [60]. Thus, we expected that FSWs who possessed high self-efficacy were more likely to form positive perceptions about the use of condoms. From this assertion, self-efficacy appears to represent an important antecedent of the perceived severity, benefits, and barriers. Our final model suggests that supports this hypothesis and self-efficacy has a direct effect on perceived severity, perceived benefits, and perceived barriers, with a positive but indirect effect on condom use behaviors. This suggests that self-efficacy may affect health behaviors indirectly through other cognitive variables, which additional studies were needed in order to confirm this hypotheses [28].

Our study has several limitations. First, despite the efforts to recruit as many participants as possible, the study was still based on a sample of convenience. Second, self-report data may result in social desirability response bias. Third, the ability to establish causal relationship is limited by using data from cross-sectional research. Fourth, the internal consistency in some of our HBM was relatively low (e.g., 0.45 for perceived susceptibility, 0.58 for perceived severity, 0.63 for perceived barriers, and 0.46 for self-efficacy). Since the value of Cronbach's alpha depends on the degree of inter-correlation and the number of items [61], the low Cronbach's alpha can be explained by the heterogeneity in HIV-related behaviors and working conditions among the FSWs in this city [19], [20], [62] and the small number of items within every dimensions in our Health belief model. It was noteworthy that we set a small number of items given that the FSWs were impatient and of inadequate literacy and given the usefulness of our investigated tool. Fifth, the number of response options was reduced from 5 to 3 in oder to help FSWs' understand the questions better, which may have influenced the model fitting in our study. Finally, perceived susceptibility was not considered in the initial hypothetical model because of low internal inconsistency between the two items.

## Conclusions

Although further investigation is still needed, the present study suggested that the HBM can be used to explore and explain the predictors of condom use behaviors in FSWs. Perceived benefits of condom use and perceived barriers to condom use are important and direct determinants of condom use among FSWs. The self-efficacy may not affect condom use behaviors directly in FSWs, but it may work by means of other variables. Future prevention interventions should be focused on the increasing in the awareness of the benefits from condom use, the reduction in the barriers to condoms use, and the improvement in self-efficacy among FSWs.

## Supporting Information

Appendix S1 **Health Belief Model (HBM) questionnaire items.**
(DOC)
