# Supplementary material for: Predictors of Condom Use Behaviors Based on the Health Belief Model (HBM) among Female Sex Workers: A Cross-Sectional Study in Hubei Province, China
Source: PLoS One. 2012 Nov 20;7(11):e49542. doi: 10.1371/journal.pone.0049542 (PMC3502447; doi:10.1371/journal.pone.0049542)
Supplement: Appendix S1 — Health Belief Model (HBM) questionnaire items. (DOC) [file pone.0049542.s001.doc]

APPENDIX

***Acculturation Items***

| *Item* | *Item wording* |
| --- | --- |
| 1 | Do you use a condom with clients in the last episode? |
| 2 | Do you use condoms with clients in the past month? |
| 3 | Do you think you are at the risk of HIV infections? |
| 4 | Do you think it’s possible to get HIV from apparently healthy clients? |
| 5 | If I had AIDS, I can not pay the cost of treatment. |
| 6 | If I had AIDS, my family and I will be discriminated by others. |
| 7 | If I had AIDS, my life will end soon. |
| 8 | Use of condoms can reduce the risk of HIV transmission. |
| 9 | Consistent and correct condom use can prevent AIDS. |
| 10 | Consistent and correct condom use can prevent STDs. |
| 11 | Consistent and correct condom use can prevent pregnancy. |
| 12 | It costs too much to buy condoms. |
| 13 | Sometimes condoms are not around, so I don’t use them. |
| 14 | If clients want non-use condoms, I will meet their requirements |
| 15 | If a client offers higher payment, I will agree to have sex with him without using condom. |
| 16 | If a client is unwilling to use condoms, can you convince him to use condoms? |
| 17 | When you have sex with clients, can you always carry condoms? |
| 18 | If a client is unwilling to use condoms, I will not have sex with them. |
